# Addressing wholesale distributor barriers to buprenorphine access: Consensus recommendations from the PhARM-OUD expert panel

**DOI:** 10.1016/j.dadr.2025.100360

**Published:** 2025-07-10

**Authors:** Tyler J. Varisco, Douglas Thornton, Taha Hussain, Hannah Fish, Joshua Bolin, David Dadiomov, Ekere J. Essien, Matthew A. Wanat, Diane Ginsburg, Jeanne Waggener, Jeffrey P. Bratberg, Bethany DiPaula, Lucas G. Hill, PhARM-OUD Working Group

**Affiliations:** a Pharmacy Addictions Research and Medicine Program, University of Texas at Austin College of Pharmacy, US; b Health Outcomes Division, University of Texas at Austin College of Pharmacy, US; c The Prescription Drug Misuse Education and Research Center, University of Houston College of Pharmacy, USA; d Department of Pharmaceutical Health Outcomes and Policy, University of Houston College of Pharmacy, USA; e The National Community Pharmacists Association, USA; f The National Association of Boards of Pharmacy, USA; g Titus Family Department of Clinical Pharmacy, The University of Southern California College of Pharmacy, USA; h Department of Pharmacy Practice and Translational Research, University of Houston College of Pharmacy, USA; i Division of Pharmacy Practice, University of Texas at Austin, College of Pharmacy, USA; j Consulting, LLC; k The University of Rhode Island College of Pharmacy, USA; l The University of Maryland College of Pharmacy, USA; m Department of Pharmacy and Therapeutics, University of Pittsburgh School of Pharmacy, USA

**Keywords:** Pharmacists, Buprenorphine, Medication systems, Drug and narcotic control

## Abstract

**Objective:**

Less than one-in-four patients with opioid use disorder receive opioid agonist treatment. This is in part due to the fact that less than 60 % of pharmacies stock and dispense buprenorphine products for the treatment of opioid use disorder (OUD). Pharmacies do not stock for many reasons but wholesale distribution remains a major barrier to buprenorphine availability. The objective of this study was to create consensus recommendations to improve wholesale distribution of buprenorphine for the treatment OUD in community pharmacies.

**Methods:**

This study involved a qualitative elicitation study, grounded in the theory of planned behavior, with seven-focus groups and 46 total pharmacists in Texas, California, and West Virginia. Results of the reflexive thematic analysis were used to create a vignette describing pharmacy-based barriers to buprenorphine supply. Non-legislative recommendations to improve buprenorphine purchase were created through a four-round Delphi study with 22 experts in psychiatry, pharmacy practice, drug distribution, and drug-policy and public comment review between June 2022 and September 2024.

**Results:**

The elicitation study demonstrated that distributor thresholds led to buprenorphine rationing, care interruptions, payer limitations, and fear of enforcement in community pharmacies. The expert panel recommended six, consensus actions that pharmacists, DEA and distributors could take to avoid further interruptions in buprenorphine availability.

**Conclusion:**

DEA and distributors can act now, without congressional intervention, to ensure that the terms of the opioid injunctive relief agreement do not impede the ability of pharmacists to provide care to persons with OUD.

## Highlights

• Distributor thresholds cause pharmacists to ration buprenorphine products.
• Pharmacists associate dispensing buprenorphine with regulatory scrutiny.
• Distributors can ease buprenorphine thresholds without legislation.
• Pharmacists should not attempt to guess thresholds but should dispense buprenorphine.

## 1 Introduction

Opioid agonist treatment reduces risk of all-cause mortality by more than 50 % in patients with opioid use disorder (OUD) yet only one in four of the nine million adults with OUD receive medication treatment (Dowell, 2024, Larochelle et al., 2018, Santo et al., 2021). Of the three Food and Drug Administration approved medications for OUD, buprenorphine is the only one that can currently be prescribed by any physician or prescriber with Drug Enforcement Administration (DEA) prescriptive authority, and then dispensed by any community pharmacy (Sen. Hassan, 2021-2022, Varisco et al., 2023). As a partial opioid agonist, buprenorphine activates opioid receptors in the central nervous system to reduce opioid craving and withdrawal symptoms without significant respiratory depression risk. Buprenorphine carries very little risk of overdose and is involved in only 2 % of opioid overdose deaths, with over 97 % of those involving another substance (Tanz et al., 2023).

The extension of pandemic era telehealth flexibilities and the Mainstreaming Addiction Treatment Act have both effectively improved access to buprenorphine prescribers (Health and Human Services, 2023, Sen. Hassan, 2021-2022). Policies that facilitate prescribing, however, will have limited effect if patients cannot fill prescriptions (Carpenter et al., 2022). Around half of all community pharmacies do not stock buprenorphine, and one in five refuse to order it (Hill et al., 2022, Weiner et al., 2023). Stigma toward persons with OUD, payer issues, and poor communication with buprenorphine providers are all barriers for buprenorphine availability (Cooper et al., 2020, Textor et al., 2022, Ventricelli et al., 2020). One of the most salient barriers to buprenorphine dispensing is the evolving drug enforcement landscape (Carpenter et al., 2022, Qato et al., 2022). Policy changes across the regulatory hierarchy intended to prevent pharmacists from dispensing high-risk opioid prescriptions have adversely affected buprenorphine access. In 2019, the Substance Use Disorder Prevention that Promotes Opioid Recovery and Treatment Act (the SUPPORT Act) amended the federal Controlled Substance Act (CSA) to require the DEA to develop a centralized system to collect the details of suspicious controlled substance orders from pharmaceutical distributors.(Rep. Walden, 2018) The SUPPORT act allows distributors to establish their own definitions for a suspicious order (Rep. Walden, 2018).

In July of 2021, the nation’s largest opioid distributors worked with the Department of Justice (DOJ) to develop an injunctive relief agreement to settle opioid-related lawsuits in most states (Department of Justice, 2022). The Federal Injunctive Relief on Opioids contained new rules and guidelines for the enforcement of provisions of the SUPPORT act and federal CSA that pertain to Suspicious Order Reporting Systems (SORS) (Department of Justice, 2022). Although the agreement maintains the ability of distributors to set their own parameters for SORS, DOJ describes specific, non-quantitative requirements for “highly diverted” controlled substance, including buprenorphine products without naloxone. The agreement also requires distributors to set ordering thresholds at the pharmacy level. These limits are usually established using algorithms that predict thresholds based on historical dispensing patterns. Thresholds are distributor-specific, resulting in varying limits between distributors for the same pharmacy. In the agreement, distributors are also barred from discussing thresholds with client pharmacies. If a pharmacy crosses a threshold, the pharmacies order is reported to DEA. Pharmacies and pharmacists often inappropriately limit buprenorphine ordering and dispensing to avoid investigation (Cooper et al., 2020; Glanz et al., 2023).

Several solutions have been suggested to avoid buprenorphine rationing, including reclassifying buprenorphine as a non-controlled substance, requiring pharmacists to dispense buprenorphine, expanding home delivery of buprenorphine products, and clearer regulatory guidance from DEA (Ostrach et al., 2021, Qato et al., 2022). If passed, the Broadening Utilization of Proven and Effective Treatment for Recovery Act, would exempt buprenorphine entirely from SORS for the remainder of the opioid public health crisis (Sen. Heinrich, 2024). While this would improve buprenorphine access, administrative action could be taken now to streamline buprenorphine wholesale without awaiting legislative action.

In fall of 2023, a group of investigators from the National Association of Boards of Pharmacy (NABP), the National Community Pharmacists Association (NCPA), and three colleges of pharmacy (the University of Houston, the University of Texas, and the University of Southern California) convened to develop consensus recommendations to improve access to medication for opioid use disorder in community pharmacies. The Pharmacy Access to Resources and Medication for Opioid Use Disorder (PhARM-OUD) Steering Committee developed a practice guideline for community pharmacists to address practice-specific barriers to buprenorphine supply. The main guidance to pharmacists, *The Pharmacy Access to Medication for Opioid Use Disorder Guideline,* contains nine recommendations and 35 supporting recommendations intended to provide solutions to community pharmacists on salient barriers to buprenorphine dispensing in their practices (Varisco et al., 2024). In recognition that broader barriers persist outside of the immediate practice environment, the PhARM-OUD panel also drafted and accepted consensus recommendations for regulators, distributors, and pharmacists to encourage policy change to address obstacles to wholesale buprenorphine distribution.

## 2 Methods

### 2.1 The elicitation study

**Fig. 1:**
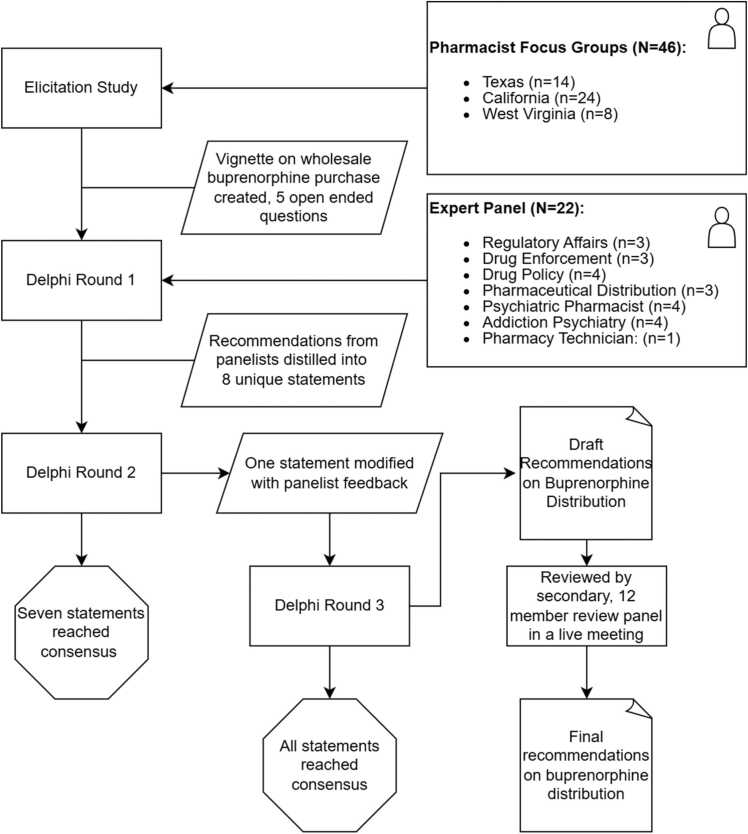
Study design used to create consensus recommendations to alleviate barriers to buprenorphine distribution.

Source: Author’s depiction of the study design

A full protocol for the elicitation and delphi components of this study was published in *The Archives of Public Health* (Varisco et al., 2024, Varisco et al., 2024)*.* Recommendations were drafted in response to vignettes developed from a literature review, focus groups, and interviews with community pharmacists in Texas, California, and West Virginia (Fig. 1). These states were selected due to differences in Medicaid expansion status and use of Medicaid wavers for addiction treatment services, differences in OUD prevalence and treatment need, and differences in socioeconomic status. Participants were purposively selected to represent both rural and urban as well as low and high socioeconomic status areas of the state. Focus groups were facilitated by a single moderator who used a semi-structured guide grounded in the Theory of Planned Behavior. The moderator guide and a description of the sampling procedure are available in the companion protocol (Varisco et al., 2024).

Following initial focus groups in Texas and California, two steering committee members (TV and MW) independently coded the transcripts, reconciled discrepancies with a third investigator (DT), and applied the final coding scheme to remaining transcripts using a reflexive, thematic approach (Braun and Clarke, 2019). Three focus groups were held in Texas, three in California, and one in West Virginia. Recruitment challenges in West Virginia led to supplemental one-on-one interviews with four pharmacists. All participants received a $200 gift card. Fourteen situational vignettes were then developed, including one focused on buprenorphine wholesale barriers.

### 2.2 Delphi study

A four-round Delphi study was conducted to generate and refine the recommendations through structured expert review (Okoli and Pawlowski, 2004). The steering committee identified experts in regulatory affairs, drug enforcement, policy, distribution, psychiatric and community pharmacy, and addiction medicine. All participants were screened by the steering committee for conflicts of interest and were offered a $1000 honorarium for their service.

In round one, panelists reviewed all fourteen vignettes and responded to open-ended questions eliciting recommendations for each. The vignette on wholesale-buprenorphine distribution featured five open-ended questions (Appendix 1, Fig. 1). The principal investigator (TV) synthesized responses into eight preliminary recommendations, which were refined with steering committee input.

In round two, participants rated each recommendation’s acceptability on a nine-point scale. Statements rated ≥ 7 by at least 70 % of respondents were retained, while others were revised and reassessed in round three based on the panelists’ suggested modifications. The final round consisted of a two-hour virtual panel meeting. Following the Delphi process, the steering committee posted draft guidance on the National Association of Boards of Pharmacy website for public comment (April 15–June 1, 2024). A review panel of 12, separate members with similar expertise convened June 11–12, 2024 at NABP headquarters in Mount Prospect, IL to incorporate public comment and assess the feasibility of recommendations to pharmacists and distributors, alike.

This study was registered with Open Science Framework (https://doi.org/10.17605/OSF.IO/6S9DY) and approved by the University of Houston IRB. Deviations from the protocol included fewer-than-planned expert panelists (22 vs. 40) and adjustments to West Virginia focus group recruitment. However, theoretical saturation was achieved in the elicitation study, and all wholesale recommendations met the pre-specified consensus threshold.

## 3 Results

### 3.1 Results of the elicitation study

A total of seven focus groups (three in Texas, three in California, and one in West Virginia) were conducted. The single focus group in West Virginia was supplemented with four, one-on-one interviews. In total, 46 pharmacists (24 in California, 14 in Texas, and 8 in West Virginia) participated. Participant characteristics are available in Table 1**.** The reflexive thematic analysis ended in the development of five major themes related to wholesale distribution: awareness of thresholds, buprenorphine rationing, care interruptions, payer limitations, and enforcement. Themes, definitions, and supporting quotes are provided in Table 2**.**

**Table 1:** Characteristics of pharmacists who participated in the elicitation focus groups and interviews (N = 46).

**Characteristic**	**N (%)**
**Sex at Birth**	
Male	13 (29 %)
Female	33 (71 %)
**Race**	
Asian	10 (21 %)
Black or African American	4 (8 %)
White	29 (63 %)
Other	3 (7 %)
**Age | Mean (SD)**	43 (11)
**State of Practice**	
California	24 (57 %)
Texas	14 (33 %)
West Virginia	8 (19 %)
**Years in Practice | Mean (SD)**	16 (10)
	
**Title**	
Pharmacist in charge	28 (60 %)
Staff pharmacist	15 (33 %)
Other	3 (7 %)
**Practice Type**	
Grocery Store (Kroger, Randall's, HEB)	6 (13 %)
Independent pharmacy	26 (57 %)
Mass merchandiser (Target, Walmart, Sam's Club, Costco)	2 (4 %)
Pharmacy chain (CVS, Walgreens)	12 (25 %)
**Frequency of Buprenorphine Dispensing**	
Less than once a month	10 (21 %)
A few times a month	12 (25 %)
A few times a week	7 (15 %)
Daily or almost daily	17 (38 %)
**Hours Worked per Week | Mean (SD)**	40 (6)

**Table 2:** Themes, definitions, and example quotes from a series of focus groups defining barriers to wholesale purchase of buprenorphine in community pharmacies.

**Theme**	**Definition**	**Example Quote**
Awareness of Thresholds	Quantitative limits that disrupt a pharmacy's ability to order either all or specific controlled substances from a distributor due to unknown and unknowable limits on controlled substance purchase set by the distributor.	*We ran a report. We know now. So the percentage is that we have to dispense 20 % of our total prescriptions, so for example, if we dispense a hundred prescriptions, 20 of them can be controlled substances. One we hit that quota, game over. You can order whatever you want. They won't ship anything.*
Buprenorphine Rationing	The act of restricting controlled substance prescriptions to certain patients by triaging the clinical needs of some patients over others.	*So now you've got your patients, your regular customers who live in town, who, you know, have cancer or whatever and you got to ensure that you've got medication for them and that's why we try to limit sometime these out of town prescriptions because we gotta make sure we have it for our regular customers."*
Care Interruptions	When thresholds interfere with the ability of a pharmacy to meet the health and medical needs of the patients that they serve.	*And then one of my sister stores actually was cut off for a month. They had hit their limit, they weren't cut off as in they were never going to get it again just for that four or five week rolling period. They had hit their max amount of order because of the location of where they're at. They're literally sharing a corner with a treatment facility and there's another one about 10 min from where they are and it just had unfortunately happened that month that they hit the limit with two weeks left in the period. So once they were out, they were out”*
Payer Limitations	When payer formularies or preferences cause pharmacies to preferentially stock some buprenorphine formulations over others.	*I don't know, it's nothing bad, but it's always made me kind of raise my eyebrow that our state Medicaid will only pay for name brand for Suboxone films unless they get something from the provider. I've recently seen that they are paying for the tablets, the generic form since there's only a generic to my knowledge of the tablets, but I've always felt like that was a little odd, which I'm going to assume is due to some contractual things with pharmaceutical providers, but that I always thought was odd, kind of restricting what we can offer to patients.”*
Enforcement	Audits and inspections from the Drug Enforcement Administration or other agencies that engender fear and stocking of buprenorphine in community pharmacies	*You know, I recently had a visit from the DEA, just a random inspection, and that [buprenorphine] was one of the key drugs they were interested in looking at and you know if they're going to be coming and you know if that's what they're interested in then that's not what I want to be dispensing obviously.*

Distributor concerns were discussed, with minimal prompting from moderators, in each focus group and interview. Pharmacists consistently described being “capped” or “hitting quotas”. Pharmacists described attempting to guess what those thresholds were:

“*We ran a report. We know now. So the percentage is that we have to dispense 20 % of our total prescriptions, so for example, if we dispense a hundred prescriptions, 20 of them can be controlled substances. One we hit that quota, game over. You can order whatever you want. They won't ship anything*.”

-*Independent community pharmacist, California*

While some pharmacists tried to guess, and work around, their thresholds, most felt that they could not guess when their buprenorphine shipments would be disrupted. Several mentioned that there was no warning and that orders were cancelled suddenly:

*“Our pharmacy actually got cut off from [DISTRIBUTOR NAME] from ordering controls, no warning, they turned into report and they were like, oh, you can't order controls. They are working to get it back, but there was no warning just as of today you can't order 'em anymore.”*

-*-Chain pharmacist, West Virginia*

A direct consequence of thresholds was the perceived need to ration care, particularly for patients living outside of their normal service area. Pharmacists mentioned that they felt the need to prioritize patients with pain over patients with addiction.

*“So now you've got your patients, your regular customers who live in town, who, you know, have cancer or whatever and you got to ensure that you've got medication for them and that's why we try to limit sometime these out of town prescriptions because we gotta make sure we have it for our regular customers."*

*-Independent Pharmacist, Texas*

While some pharmacies rationed care, others made more deliberate effort to meet the treatment needs of OUD patients by prioritizing buprenorphine over other opioids. The inflexibility of distributor thresholds made it impossible for them to dispense an adequate amount of buprenorphine:

*“They're literally sharing a corner with a treatment facility and there's another one about 10 min from where they are and it just had unfortunately happened that month that they hit the limit with two weeks left in the period. So once they were out, they were out.”*

*-Chain pharmacist, West Virginia*

Some state Medicaid plans receive contractual rebates from the manufacturer of brand name buprenorphine/naloxone buccal films through the Medicaid Drug Rebate Program (MDRP). Pharmacists felt that MDRP limited availability generic buprenorphine/naloxone buccal films, placing commercially insured patients, unable to fill prescriptions for brand-name buprenorphine, at a disadvantage:

*“When Medi-Cal decides to force you to buy brand, that doesn’t take into consideration pharmacy's contracts with their wholesalers that require they purchase enough generics to get a specific rebate… And if they're required to buy more brands, it throws off their generic compliance ratio, they don't get as good of a rebate.”*

*-Independent Pharmacist, California*

Finally, pharmacists discussed their experiences with DEA and distributor investigations. Of note, pharmacists mentioned that DEA investigators directly requested to review buprenorphine prescriptions dispensed. Another pharmacist described an audit by their distributor where the investigator asked to review buprenorphine records. The investigator described that this was due to recent regulatory pressure from DEA.

*“You know, I recently had a visit from the DEA, just a random inspection, and that [buprenorphine] was one of the key drugs they were interested in looking at and you know if they're going to be coming and you know if that's what they're interested in then that's not what I want to be dispensing obviously. Same thing with our wholesaler”*

*-Independent Pharmacist, California*

### 3.2 The Delphi study

Twelve of the 22 members of the expert panel were healthcare practitioners (either physicians or pharmacists), eight had expertise in drug policy, four in wholesale drug distribution, six were current or former members of boards of pharmacy, and three were experts in drug enforcement or litigation related to drug enforcement. The participants are named as members of the PhARM-OUD Working Group, above.

In the first round of the Delphi panel, participants provided 84 unique statements in response to the five items in the vignette on wholesale purchase. In general, participants urged measured caution and generous discretion with procedures for monitoring buprenorphine wholesale purchases. While many participants felt that buprenorphine should be exempted from SOR programs all together, some felt that SOR programs should prioritize flexibility for pharmacies that dispense higher volumes of buprenorphine in response to clinically necessary prescriptions. No participants expressed an interest in further restricting buprenorphine supply.

The 84 statements were coded by the steering committee and condensed into eight, unique statements for evaluation in round two (Appendix 1, Supplemental Table 1). In round two, seven of the eight statements reached consensus. One statement, *“To minimize the risk of regulatory scrutiny, investigation, or wholesaler sanction, it is advisable for pharmacists to concentrate on enhancing their relationship with their current wholesaler rather than ordering buprenorphine or controlled substances from multiple wholesalers,”* was only rated as acceptable by 59 % of respondents. The statement was revised, based on panelist feedback to read: “*Ordering buprenorphine from multiple wholesalers may increase risk of regulatory scrutiny. Buprenorphine orders, particularly in the independent pharmacy setting, should be concentrated with one wholesaler.”* In round three, panelists were asked to re-evaluate the revised statement and were given the opportunity to read the suggested modifications made by other panelists. Here, 77.2 % of participants rated this statement as appropriate. As all eight statements reached consensus, none were modified further in the live fourth round.

### 3.3 Revision of the consensus statements

In the review of public comments hosted by NABP, two statements were removed from the original eight. One, “*Increased demand for buprenorphine stemming from the passage of the Mainstreaming Addiction Treatment Act has created an urgent need for pharmaceutical wholesalers to revise the parameters of buprenorphine suspicious order monitoring algorithms,”* was believed to be a statement of fact rather than an actionable recommendation. It was also felt that the spirit of this recommendation was adequately captured in other, more direct recommendations. Another *“Pharmaceutical wholesalers should transparently communicate the terms of buprenorphine suspicious order monitoring programs to pharmacies and provide written notification to pharmacies in advance of restricting further buprenorphine purchase to prevent patients from losing access to medication for opioid use disorder,”* was thought to be infeasible within the confines of the Federal Injunctive Relief Agreement. The review panel also encouraged the steering committee to revise all recommendations into shorter, more direct statements to improve readability and to replace the term “wholesaler” with “distributor” throughout. The revised final statements are below in Table 3.

**Table 3:** Revised, final recommendations from a 22-member expert panel to support actions to improve access to buprenorphine in community pharmacies.

Buprenorphine orders should be concentrated with one distributor rather than multiple.
Pharmacists should not decline to dispense buprenorphine based on speculative concerns about potential distributor-controlled substance purchasing restrictions
The Drug Enforcement Agency (DEA) should provide guidance to pharmaceutical distributors to ensure that Suspicious Order Reporting Systems (SORS) do not interfere with a pharmacy’s ability to dispense buprenorphine.
Pharmaceutical distributors should urgently revise the parameters of their buprenorphine SORS with or without input from DEA.
SORS should be designed by distributors to monitor buprenorphine orders in isolation from other controlled substances.
Pharmaceutical distributors should clarify procedures for threshold change requests and ensure that threshold change requests are urgently approved for buprenorphine orders.

## 4 Discussion

Through focus groups with pharmacists in three states and a Delphi study, we identified six actionable solutions to improve buprenorphine distribution. Importantly, the proposed solutions can be implemented now without congressional action. It is worth noting that these recommendations do not apply solely to distributors but to the Drug Enforcement Administration and pharmacists as well. The expert panel indicated that pharmacist actions had little bearing on buprenorphine distribution but did recommend two pharmacy-based solutions. First, pharmacists were recommended to concentrate purchasing with one distributor. Purchasing as many medications as possible from one distributor, including buprenorphine, is expected to improve the pharmacist’s ratio of controlled substances to non-controlled substances, a key SOR metric (Department of Justice, 2022). Restricting purchasing to one distributor may not be financially viable to all pharmacies, particularly smaller independent pharmacies. Pharmacists already cite limited reimbursement as a significant barrier to buprenorphine dispensing and concentrating orders with one distributor precludes value-based purchasing (Freeman et al., 2024). In many ways, this proposed action is unlikely to be acceptable to pharmacies who already struggle to receive adequate reimbursement for the services they provide (United States House Committee on Oversight and Accountability, 2024). Reforming distributor practices, especially those used to set SOR thresholds, through DEA guidance and collaboration would be less likely to cause financial harm to pharmacies.

The panel also recommended that pharmacists stop attempting to guess buprenorphine thresholds. Rather, pharmacists should continue to dispense buprenorphine until they hit their threshold and then work with their distributor to submit a threshold change request. If pharmacists assume that they have met, or will soon meet, their threshold, they are likely to decline to fill otherwise legitimate prescriptions, jeopardizing patient health and safety. As pharmacists often use internal procedures to avoid surpassing thresholds and there is currently no mechanism to collect data on threshold breaches, it is difficult to know how frequently threshold breeches would occur were pharmacists to dispense every buprenorphine prescription brought to their pharmacy. The American Society of Addiction Medicine is collecting reports of buprenorphine prescription issues in an online portal (American Society of Addiction Medicine, 2024). National pharmacy organizations should provide a similar portal for pharmacists to report threshold breaches. Allowing pharmacists to anonymously report the circumstances that lead to threshold breeches would provide needed evidence to refine SOR programs to improve buprenorphine access.

In the elicitation study, pharmacists universally indicated that they had little control, or knowledge, of how buprenorphine thresholds were. This places the onus on regulatory agencies and distributors to ensure that policies originally intended to prevent overdose deaths by restricting opioid supply do not worsen opioid deaths by interfering with pharmacists’ ability to dispense medically necessary prescriptions. This expert panel recommended that the DEA provide specific guidance to distributors to ensure that pharmacists can continue to fill medically necessary prescriptions. Even though this is not DEA’s direct responsibility, neither the injunctive relief agreement nor DEA’s scope of authority would bar DEA from providing specific guidance to distributors (Khail et al., 2024). The expert panel did not suggest specific thresholds as this would be impossible without an understanding of the current design and parameters of SOR algorithms. Still, 90.9 % of panelists agreed with improved DEA guidance.

To achieve this goal, the panel recommended that SORS should monitor buprenorphine in isolation of other controlled substances. For instance, if a pharmacy passes their hydrocodone threshold in any given month, this should not interfere with their ability to order buprenorphine. Despite language in the injunctive relief agreement instructing distributors to set drug family-specific thresholds, participants in the elicitation study consistently recounted losing their ability to order all opioids if they crossed a single-drug threshold. If buprenorphine remains entangled with other controlled substances, then pharmacists will continue engage in rationing. If pharmacies lose their ability to purchase buprenorphine when they cross thresholds for other controlled substances, this depresses access to treatment in a way that is inconsistent with the intended public health purpose of multistate opioid litigation. Second, the panel recommended that buprenorphine distributors should consider revising SOR parameters even without the benefit of DEA guidance. The injunctive relief allows distributors to set, review, and modify the parameters of SORS on their own (Department of Justice, 2022). Even if DEA does not provide direct guidance, distributors could act in the interest of public health to introduce more leniency into current buprenorphine thresholds.

The panel also recommended that distributors take action now to simplify threshold change request procedures for buprenorphine. If a pharmacy can substantiate that they are dispensing more buprenorphine prescriptions due to a new relationship with an opioid treatment center or due to a new commitment to serve telehealth patients, or for many other legitimate reasons, then there is no real reason that the distributor should refuse that request assuming the pharmacy can provide supporting evidence. Additionally, if a pharmacy crosses a buprenorphine threshold, distributors should provide a mechanism for pharmacists to quickly return to dispensing. In the elicitation study, pharmacists described waiting four or more weeks after crossing a threshold to be able to purchase controlled substances. As less than 60 % of pharmacies dispense buprenorphine to begin with, there is a high likelihood a specific pharmacy is the only pharmacy near the patient that dispenses buprenorphine (Hill et al., 2022, Weiner et al., 2023). Even short disruptions in treatment of two weeks or less increases risk of mortality for patients in treatment (Cornish et al., 2010). It is highly unlikely that the time required for a distributor to perform a record review or on-site audit in response to a buprenorphine threshold change request is worth the risk to patients’ lives.

Despite the strength of the statements generated, this study did have some limitations. First, delays in focus group recruitment led to slightly more fragmented data collection than initially planned. High levels of agreement between pharmacists in all states, however, suggests that the results were trustworthy (Glaser and Strauss, 2006). Second, 22 experts were recruited to the Delphi panel rather than the 40 originally planned. This is an acceptable number of experts and is consistent with other Delphi studies (Akins et al., 2005). The panel represented a diverse array of perspectives and was able to efficiently generate and evaluate consensus statements. Finally, the inherent nuance of buprenorphine distribution made it difficult to assemble comprehensive evidence evaluating the association between limits on buprenorphine distribution and treatment outcomes.

## 5 Conclusions

Barriers to buprenorphine distribution are a tragic consequence of efforts to prevent inappropriate opioid prescribing. DEA and DOJ must provide transparent guidance to distributors to establish reasonable buprenorphine thresholds that align regulation with patient care. Legislative action could be taken to exempt buprenorphine from SORS but this study provided immediate solutions that are possible without congressional action. Addressing these barriers is critical to reducing the risk of opioid-related harm in communities. A coordinated effort by regulatory agencies, distributors, and pharmacists is essential to ensure timely and equitable access to buprenorphine.

## CRediT authorship contribution statement

**Lucas G. Hill:** Writing – review & editing, Writing – original draft, Resources, Investigation, Funding acquisition, Conceptualization. **Douglas Thornton:** Writing – review & editing, Writing – original draft, Investigation, Funding acquisition, Formal analysis, Conceptualization. **Bethany DiPaula:** Writing – review & editing, Writing – original draft, Investigation. **Tyler J. Varisco:** Writing – review & editing, Writing – original draft, Methodology, Investigation, Funding acquisition, Formal analysis, Data curation, Conceptualization. **Hannah Fish:** Writing – review & editing, Writing – original draft, Funding acquisition, Conceptualization. **Taha Hussain:** Writing – review & editing, Writing – original draft, Formal analysis. **David Dadiomov:** Writing – review & editing, Writing – original draft, Investigation, Funding acquisition, Conceptualization. **Joshua Bolin:** Writing – review & editing, Writing – original draft, Funding acquisition, Conceptualization. **Matthew A. Wanat:** Writing – review & editing, Writing – original draft, Investigation, Formal analysis, Conceptualization. **Ekere J. Essien:** Writing – review & editing, Writing – original draft, Funding acquisition, Formal analysis, Conceptualization. **Jeffrey P. Bratberg:** Writing – review & editing, Writing – original draft, Conceptualization. **Jeanne Waggener:** Writing – review & editing, Writing – original draft, Funding acquisition, Conceptualization. **Diane Ginsburg:** Writing – review & editing, Writing – original draft, Funding acquisition, Conceptualization.

## Author disclosures

This work was funded by the Foundation for Opioid Response Efforts (FORE). The views and conclusions contained in this document are those of the authors and should not be interpreted as representing the official policies or stance, either expressed or implied, of FORE. FORE is authorized to reproduce and distribute reprints for Foundation purposes notwithstanding any copyright notation hereon.

## Declaration of Competing Interest

The authors declare the following financial interests/personal relationships which may be considered as potential competing interests: Tyler Varisco reports financial support was provided by Foundation for Opioid Response Efforts. Kelly Clark reports a relationship with Bicycle Health Inc that includes: consulting or advisory. Scott Weiner reports a relationship with Bicycle Health Inc that includes: equity or stocks. Scott Weiner reports a relationship with Vertex Pharmaceuticals Incorporated that includes: board membership. If there are other authors, they declare that they have no known competing financial interests or personal relationships that could have appeared to influence the work reported in this paper.

## Footnotes

Appendix A Supplementary data associated with this article can be found in the online version at doi:10.1016/j.dadr.2025.100360.
