# Supplementary material for: Addressing wholesale distributor barriers to buprenorphine access: Consensus recommendations from the PhARM-OUD expert panel
Source: Drug Alcohol Depend Rep. 2025 Jul 10;16:100360. doi: 10.1016/j.dadr.2025.100360 (PMC12301831; doi:10.1016/j.dadr.2025.100360)
Supplement: Supplementary file 1 — Supplementary material [file mmc1.docx]

**Supplemental Figure 1:** A vignette and seven, free response items intended to elicit solutions to barriers that interfere with pharmacists’ ability to purchase buprenorphine from pharmaceutical distributors.

**Wholesale buprenorphine purchase:**
Pharmacists report that purchasing buprenorphine from wholesale vendors is complicated by the fact that buprenorphine sales are subject to the algorithmic limits of wholesaler suspicious order reporting programs. In addition to limits related to the amount of buprenorphine purchased, pharmacists report that dispensing buprenorphine skews the controlled substance to non-controlled drug ratio dispensed in their pharmacy making it difficult for them to purchase other controlled substances. Pharmacists in states with Medicaid plans that have negotiated rebate prices on brand name buprenorphine products also report that dispensing buprenorphine to Medicaid patients skews their brand to generic purchase ratios leading wholesalers to increase their generic drug prices. These pharmacists also report that requiring brand name products makes it difficult for them to stock generic buprenorphine. Respond to the following items based on this vignette.

**Items:**

1. Should buprenorphine orders be subject to the same suspicious order reporting requirements as other schedule III controlled substances? Please justify your answer.
2. What criteria can pharmacists use to determine if a buprenorphine order is likely to be flagged as a suspicious order?
3. How can pharmacists and pharmacy owners modify their wholesale purchasing strategy to ensure that they can continue to purchase buprenorphine?
4. What policy changes would ensure that pharmacists can continue to purchase buprenorphine from their wholesalers?
5. What alternatives, if any, do state Medicaid plans have to promote access to buprenorphine in pharmacies without relying on the Medicaid Drug Rebate Program?

*Appendix 1: Supplemental Tables and Figures*

| **Supplemental Table 1:** Draft recommendations to address barriers to buprenorphine wholesale generated by a 22 member Delphi panel. |
| --- |
| **Drafted Statement** |
| The Controlled Substance Act mandates that pharmaceutical wholesalers disclose suspicious orders to the Drug Enforcement Administration. As long as buprenorphine remains a controlled substance, buprenorphine orders must be monitored through suspicious order monitoring programs. As the parameters of suspicious order monitoring programs are established by the pharmaceutical wholesalers rather than by the Drug Enforcement Administration, pharmaceutical wholesalers and the drug enforcement administration should take steps to ensure that suspicious order monitoring programs do not interfere with a pharmacy's ability to dispense buprenorphine in response to legitimate prescriptions. |
| Suspicious order monitoring programs designed by wholesalers should be designed to monitor buprenorphine in isolation of other controlled substances. A pharmacy's ability to purchase buprenorphine should not depend on their purchases of other controlled substances. |
| Increased demand for buprenorphine stemming from the passage of the Mainstreaming Addiction Treatment Act has created an urgent need for pharmaceutical wholesalers to revise the parameters of buprenorphine suspicious order monitoring algorithms. |
| The Drug Enforcement Administration should provide public, transparent guidance to pharmaceutical wholesalers to improve the quality and consistency of buprenorphine suspicious order monitoring programs across pharmaceutical wholesalers. |
| Pharmaceutical wholesalers should transparently communicate the terms of buprenorphine suspicious order monitoring programs to pharmacies and provide written notification to pharmacies in advance of restricting further buprenorphine purchase to prevent patients from losing access to medication for opioid use disorder. |
| As suspicious order monitoring programs are designed to identify deviations from historical purchasing patterns, wholesalers should establish a process that enables pharmacies to explain and appeal decisions made due to appropriate and clinically justifiable deviations from their historical purchasing behavior. |
| Pharmacists should not decline to dispense buprenorphine based on speculative concerns about potential wholesaler controlled substance purchasing restrictions. It is not within their ability to reliably predict such restrictions. |
| To minimize the risk of regulatory scrutiny, investigation, or wholesaler sanction, it is advisable for pharmacists to concentrate on enhancing their relationship with their current wholesaler rather than ordering buprenorphine or controlled substances from multiple wholesalers. |
